# Supplementary material for: An open-source framework for large-scale, flexible evaluation of biomedical text mining systems
Source: J Biomed Discov Collab. 2008 Jan 29;3:1. doi: 10.1186/1747-5333-3-1 (PMC2276192; doi:10.1186/1747-5333-3-1)
Supplement: Additional file 1 — Evaluation platform source code. The additional file contains the software evaluation framework discussed in this paper. The most current release can be downloaded from SourceForge.net [1]. See the accompanying README file for instructions on installation and use. Also included in the distribution is a collection of UIMA wrappers for some commonly used BioNLP tools and annotated corpora. [file 1747-5333-3-1-S1.zip › bionlp_uima_repository-1.0.1/docs/api/edu/uchsc/ccp/util/external/penn/PennBioTaggerEISystem.html]

PennBioTaggerEISystem (Center for Computational Pharmacology Util/UIMA API)


|  |  |  |
| --- | --- | --- |
| **Overview** \| **Package** \| **Class** \| **Tree** \| **Deprecated** \| **Index** \| **Help** | |  |
| PREV CLASS   **NEXT CLASS** | **FRAMES**    **NO FRAMES**     **All Classes** |  |
| SUMMARY: NESTED \| FIELD \| CONSTR \| METHOD | DETAIL: FIELD \| CONSTR \| METHOD |  |


---


## edu.uchsc.ccp.util.external.penn Class PennBioTaggerEISystem

```
java.lang.Object
  edu.uchsc.ccp.util.external.penn.PennBioTaggerEISystem
```

**All Implemented Interfaces:**: EISystem

---

``` public class PennBioTaggerEISystem extends Object implements EISystem ```

---

| **Field Summary** | |
| --- | --- |
| `static String` | `TAGGER_TYPE_GENE` |
| `static String` | `TAGGER_TYPE_MALIGNANCY` |
| `static String` | `TAGGER_TYPE_VARIATION` |


**Constructor Summary**

`PennBioTaggerEISystem(String taggerType,
String entityModelFile,
String tokenizerModelFile)`
  
          Initialize a new tagger.


| **Method Summary** | |
| --- | --- |
| `List<TextAnnotation>` | `getEntitiesFromString(String inputText, String inputDocumentID)`             Find entities in a particular String of text. |

**Methods inherited from class java.lang.Object**

`clone, equals, finalize, getClass, hashCode, notify, notifyAll, toString, wait, wait, wait`

**Field Detail**

### TAGGER\_TYPE\_GENE

```
public static final String TAGGER_TYPE_GENE
```

**See Also:**: Constant Field Values

---


### TAGGER\_TYPE\_VARIATION

```
public static final String TAGGER_TYPE_VARIATION
```

**See Also:**: Constant Field Values

---


### TAGGER\_TYPE\_MALIGNANCY

```
public static final String TAGGER_TYPE_MALIGNANCY
```

**See Also:**: Constant Field Values


**Constructor Detail**

### PennBioTaggerEISystem

```
public PennBioTaggerEISystem(String taggerType,
                             String entityModelFile,
                             String tokenizerModelFile)
```

:   Initialize a new tagger. Tagger can either be for genes, variations, or malignancies.

    **Parameters:**: `taggerType` -: `entityModelFile` -: `tokenizerModelFile` -


**Method Detail**

### getEntitiesFromString

```
public List<TextAnnotation> getEntitiesFromString(String inputText,
                                                  String inputDocumentID)
```

:   Find entities in a particular String of text.

    :   **Specified by:**: `getEntitiesFromString` in interface `EISystem`


---


|  |  |  |
| --- | --- | --- |
| **Overview** \| **Package** \| **Class** \| **Tree** \| **Deprecated** \| **Index** \| **Help** | |  |
| PREV CLASS   **NEXT CLASS** | **FRAMES**    **NO FRAMES**     **All Classes** |  |
| SUMMARY: NESTED \| FIELD \| CONSTR \| METHOD | DETAIL: FIELD \| CONSTR \| METHOD |  |


---
